# A case report of *Cedecea davisae* infection in a patient with chronic kidney disease in Saudi Arabia

**DOI:** 10.1016/j.idcr.2025.e02309

**Published:** 2025-07-01

**Authors:** Hani S. Al-Jahdali, Huda S. Al Ghamdi, Bothayna I. Saleh, Mohammed F. Merza, Bushra H. Alqurashy, Layali S. Abdurabo, Zahra A. Albishi, Rasha Binmahfouz

**Affiliations:** a Medical Department, King Abdulaziz Hospital and Oncology Center, Jeddah, Saudi Arabia; b Microbiology Department, King Abdulaziz Hospital and Oncology Center, Jeddah, Saudi Arabia; c Clinical Pathology Department, Faculty of Medicine, Al Azhar University, Cairo, Egypt; d Laboratory Department, King Abdulaziz Hospital and Oncology Center, Jeddah, Saudi Arabia

**Keywords:** *Cedecea davisae*, Gram-negative, Immunocompromised, Chronic renal disease

## Abstract

*Cedecea,* a genus belonging to the family Enterobacteriaceae, are uncommonly attributed as causative agents of human infections. *Cedecea davisae* is considered as an opportunistic pathogen that was isolated from a different acute infection in advanced-aged patients with many comorbid diseases or from immunocompromised hosts. Fourteen cases of proved *C. davisae* infections were published up till now. We isolated *C. davisae* from chronic kidney disease patient who presented with purulent discharge from dialysis catheter, and the result of microbiology culture and identification on automated microbiology identification system, MicroScan WalkAway using NBC50 panel revealed *C. davisae* as a causative agent of catheter related infection. The patient improved on receiving antibiotic treatment according to culture and sensitivity results. Awareness of the clinical significance of this low incidence bacterial infection and their antibiotic resistance and sensitivity pattern is crucial in the medical field.

## Highlights

• Cedecea davisae is an opportunistic pathogen in immunocompromised hosts.
• Cedecea davisae can cause dialysis catheter-related infection.
• Awareness of C. davisae's low incidence, resistance, and sensitivity is important.

## Introduction

The genus *Cedecea* is a Gram-negative, non-encapsulated, facultative anaerobic bacterium that had been reported in only a few cases with varying clinical presentations, drug susceptibility, and treatment. In 1981, *Cedecea* was designated as a separate genus of the Enterobacteriaceae family as it was phenotypically distinct from the other members of the family and given its name [1].

Its transmission mechanism is unknown, even though it could be related to the environment, hospital contacts, host immunity status, use of antimicrobials, or other factors that will be discovered. The *Cedecea* genus consists of five species: *Cedecea davisae* (*C. davisae*), *Cedecea neteri*, *Cedecea lapagei*, and two others unnamed species [2].

*C. davisae* causes a wide spectrum of acute infections in immunocompromised hosts, from pneumonia and bacteremia to oral ulcers and dialysis-related peritonitis [3]. While *Cedecea* infections are reported infrequently in the literature, documented clinical cases of this emerging opportunistic human pathogen, albeit rare, may be due to misdiagnosis or underreporting [4].

Clinical strains of *C. davisae* exhibit variable resistance to various antimicrobial agents, including ampicillin, ampicillin-sulbactam, cefazolin, cephalothin, cefoxitin, and colistin[3]. We aimed in this study to document a case report of *C. davisae* from catheter discharge in ERSD patient attending King Abdullah bin Abdulaziz Hospital, Jeddah, KSA for peritoneal dialysis and to review other documented case reports of patients infected with *C. davisae* as published in literature.

## Case report

**Fig. 1:**
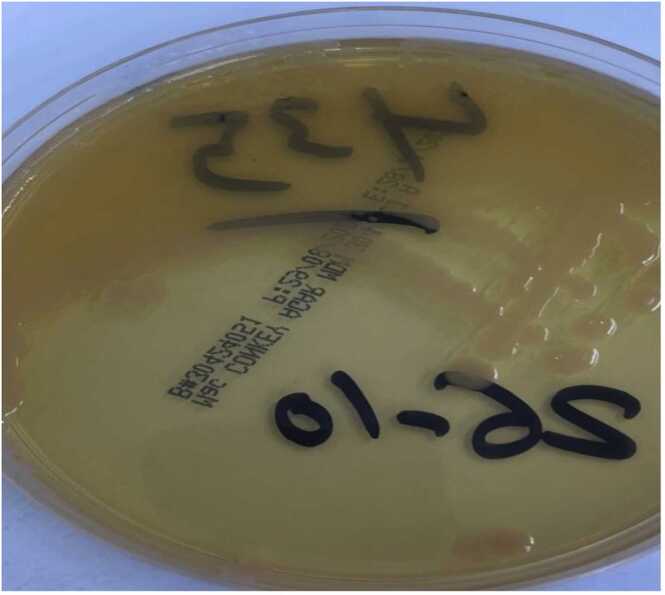
*C. davisae* colonial morphology on MacConkey agar.

A 30-year-old female with diabetes mellitus type 2, hypertension, hypothyroidism, and end-stage kidney disease has been on peritoneal dialysis since February 6, 2022. Patient presented to the peritoneal dialysis clinic on November 22, 2023, complaining of peritoneal dialysis catheter exit-site discharge for 1 day, with no history suggesting peritonitis and no signs of peritoneal dialysis catheter tunnel collection. The patient's vital signs were stable, afebrile, and patient was conscious, alert, and oriented on examination. By inspection, there was mild bloody discharge from the peritoneal dialysis catheter, and no hotness, redness, tenderness, or swelling on palpation of the catheter. A catheter swab was sent to the microbiology laboratory for culture and sensitivity, and empiric antibiotic amoxicillin 500 mg orally once daily was started. The swab was streaked on 5 % sheep blood agar, Chocolate agar, MacConkey agar and Sabaurude agar, and incubated under aerobic and anaerobic conditions at 37°C for 48 hrs. After 24 h, growth of pure mucoid, non-lactose fermenting (pale yellow) colonies was observed on MacConkey agar (Fig. 1).

The isolate was non-haemolytic on blood agar Oxidase negative. Gram stain direct from the sample swabs and from the grown colonies showed Gram-negative bacilli. Full bacterial identification and minimal inhibitory concentration (MIC) for different antibiotics were performed, according to the manufacturer’s recommendations using a fully automated microbiology identification system, MicroScan WalkAway (Dade Behring INC. West Sacramento, CA, USA) using NBC50. Quality control strains included *Pseudomonas aeruginosa* ATCC 27853 and *Escherichia coli* ATCC 25922. Antibiotic sensitivity results were interpreted according to CLSI criteria. Identification result showed an organism identified as *C. davisae* that was sensitive to Amikacin, Ciprofloxacin, Gentamicin, Meropenem, Trimethoprim/Sulfamethoxazole and Levofloxacin, and resistant to Ampicillin, Augmentin, Ceftriaxone and Tazobactam. The patient was admitted to King Abdullah bin Abdulaziz Hospital, where amoxicillin was discontinued and replaced with intravenous meropenem 500 mg once daily. The catheter was removed on December 4, 2023, and its tip was sent to the microbiology department. Culture results confirmed the presence of *C. davisae*, and meropenem treatment was continued for 14 days. A new peritoneal dialysis catheter was inserted on February 7, 2024. Swabs from the new catheter showed no bacterial growth. The patient was clinically stable and discharged.

The study was performed after approval of the Research and Studies Department – Jeddah Health Affairs Institutional Review Board (IRB) with KACST, KSA. Registration number KSA: H-02-J-002 and Approval Number: A02013.

## Discussion

*Cedecea* are Gram-negative, oxidase negative bacilli that include 5 species. This genus was designated by the Centers for Disease Control (CDC) in 1981 as a separate genus in the Enterobacteriaceae family. Strains of *Cedecea* resemble the strains of Serratia because they are lipase positive and resistant to colistin and cephalothin, but unlike Serratia they do not hydrolyze gelatin [5]. Since its discovery in 1981, a total of 14 published cases have reported the isolation of *Cedecea davisae* from various clinical samples. These include blood [4], [5], [6], [7], [8], urine, [9], [10], oral and cutaneous ulcers [6], [11], a scrotal abscess [12], sputum [13], [14], mucocele [15] and tissue [16]. In the tissue case, *C. davisae* was isolated from a hand trauma debridement and showed resistance to multiple antibiotics due to AmpC β-lactamase overproduction (Table 1).

**Table 1:** *Cedecea davisae* cases list as in literature.

Study	Age/sex	Country	Comorbidity	Infection/sample	Antibiotic resistance	Antibiotic treatment
1981 Bae et al.	76/M	USA	Ischemic heart disease	Bacteremia/sputum	Cephalothin	Cephalexin/Improved
	41/F	USA	Diabetes, congestive heart failure	Bacteremia/sputum	Cephalothin	Cefazolin/Improved
1983 Bae & Sureka	50/M	USA	Chronic heart disease, alcoholic, hepatitis	Scrotal Abscess	Ampicillin, Cephalothin, Cefamandole, Cefoxitin, Chloramphenicol	Tetracycline/ Improved
1986 Perkins et al.	70/F	USA	Heart disease, bronchitis, chronic obstructive pulmonary disorder	Bacteremia/blood	Ampicillin, Cephalothin, Cefoxitin,	Mezlocillin, Gentamicin and Clindamycin/ Improved
2008 Dalamaga et al.	67/M	Greece	Diabetes	Leg ulcer, blood	Cephalothin, Cefuroxime sodium, Cefoxitin, Ampicillin, Piperacillin, Nitrofurantoin	Cefotaxime, Amikacin
2010 Mawardi et al.	42/M	USA	Diabetes, hypertension, kidney transplantation	Superinfection of a sirolimus associated oral ulcer	Cefazolin	Ciprofloxacin
2012 Akinosoglou et al.	54/M	Greece	Sigmoid colon cancer	Blood	Tobramycin	Gentamycin
2012 Ismael et al.	20/F	USA	Diabetes, cystic fibrosis	Sputum	All tested β-lactams, Aminoglycosides, Fluoroquinolones, Tigecycline	Trimethoprim/Sulfamethoxazole
2013 Peretz et al.	77/F	Israel	Chronic kidney disease, diabetic nephropathy, hypertension	Blood, Permacat catheter tip	Ampicillin, Ampicillin/Sulbactam and Cefazolin	Ciprofloxacin and Ceftazidim
2014 Ammenouche et al.	77/F	France	Hemorrhagic shock, retroperitoneal bleeding	Urine	Ticarcillin, Cephaloridine, Cephalothin, Cefoxitin, Cefotaxime, Ceftazidime, Aztreonam, Ertapenem	Ciprofloxacin
2015 Bayir et al.	57/F	Turkey	Hypertension, diabetes, chronic kidney disease, atrophic rhinitis with mucocele	Mucocele	No resistance	Levofloxacin
2018 Agbonlahor et al.	76/M	Nigeria	Prostatectomy	Urine	Augmentin, Gentamicin, Ofloxacin, Cefixime, Ciprofloxacin, Ceftazidime and Cefuroxime	Imipenem
2019 Kanakadandi et al.	41/F	USA	Biliary cancer, Cholecystostomy	Blood	Ampicillin, Ceftriaxone, Cefuroxime	Ciprofloxacin, Metronidazole
2022 Notter et al.	33/M	Switzerland	Curatively treated seminoma	Post trauma soft tissue infection /Tissue	Ceftriaxone, Ceftazidime, Piperacillin-Tazobactam, Cefepime	Meropenem

To the best of our knowledge, from 2022 to date, our case of *C. davisae* is considered to be the 15th to be published worldwide and the first *C. davisae* reported in KSA, and the third *Cedecea* species isolated in KSA. Only two cases of the other species of *Cedecea* reported in KSA, in 2017 Arishi et al. [17] recovered *C. neteri* from a case of neonatal peritonitis in a male preterm neonate who developed the infection after his colon perforated due to necrotizing enterocolitis and in 2022, Hakami et al. [18] reported isolation of *C. lapagei* from urinary tract infection patient. Infection with *C. davisae* was found to be worldwide, not confined to certain locality or race with an extended clinical spectrum of infections. *C. davisae* was most frequently recovered from blood (6 isolates; 46 %) and sputa (3 isolates; 23 %). Ten (67 %) of the 15 patients with confirmed *C. davisae* infections were ≥ 50 years of age, and most were severely immunocompromised with multiple comorbid diseases [3], as we found the rate of *C. davisae* infections is prevalent in patients with hypertension and/or cardiac disease (n = 8), diabetes mellitus (n = 4), cancer (n = 3), chronic kidney disease (n = 2), cystic fibrosis (n = 1), a transplanted kidney (n = 1), and chronic obstructive pulmonary disease (n = 1). Although rare, these clinical cases clearly point to the ability of *C. davisae* to colonize and infect different parts of the human host, resulting in a diverse species with variable antibiotic susceptibility pattern. Fortunately, in all cases of *C. davisae* infection, all patients recovered with appropriate antibiotic treatment. However, it was noticed that there is an increasing spectrum of resistance over the time, from its discovery.

## Conclusion

Accurate identification and sensitivity testing of *Cedecea* isolates are important because, although infections are infrequent, the organism is widely distributed in the environment and can complicate underlying conditions in immunocompromised patients. The high incidence of multi-drug resistant strains further complicates diagnosis and management. Epidemiological studies are recommended to trace the sources and modes of infection and to support the isolation and diagnosis of these bacteria, especially in low-resource laboratories.

## Ethical approval

The study was performed after approval of the Research and Studies Department – Jeddah Health Affairs Institutional Review Board (IRB) with KACST, KSA. Registration number KSA: H-02-J-002 and Approval Number: A02013. Written informed consent was obtained from the patient after explaining the purpose of the report. The patient agreed to share their medical information and images, with the understanding that personal details would be kept confidential.

## CRediT authorship contribution statement

**Rasha Binmahfouz:** Writing – review & editing. **Abdurabo Layali S:** Visualization, Validation. **Albishi Zahra A:** Writing – original draft. **Merza Mohammed F:** Resources, Project administration. **Alqurashy Bushra H:** Supervision, Software. **Alghamdi Huda:** Data curation, Conceptualization. **Saleh Bothayna I:** Methodology, Investigation. **Al-Jahdali Hani S:** Funding acquisition, Formal analysis.

## Funding

This research did not receive any specific grant from funding agencies in the public, commercial, or not-for-profit sectors.

## Declaration of Competing Interest

The authors declare that they have no known competing financial interests or personal relationships that could have appeared to influence the work reported in this paper.
